# Supplementary figures and images for: Reliability of the weight-bearing ankle dorsiflexion range of motion measurement using a smartphone goniometer application
Source: PeerJ. 2021 Sep 22;9:e11977. doi: 10.7717/peerj.11977 (PMC8464192; doi:10.7717/peerj.11977)

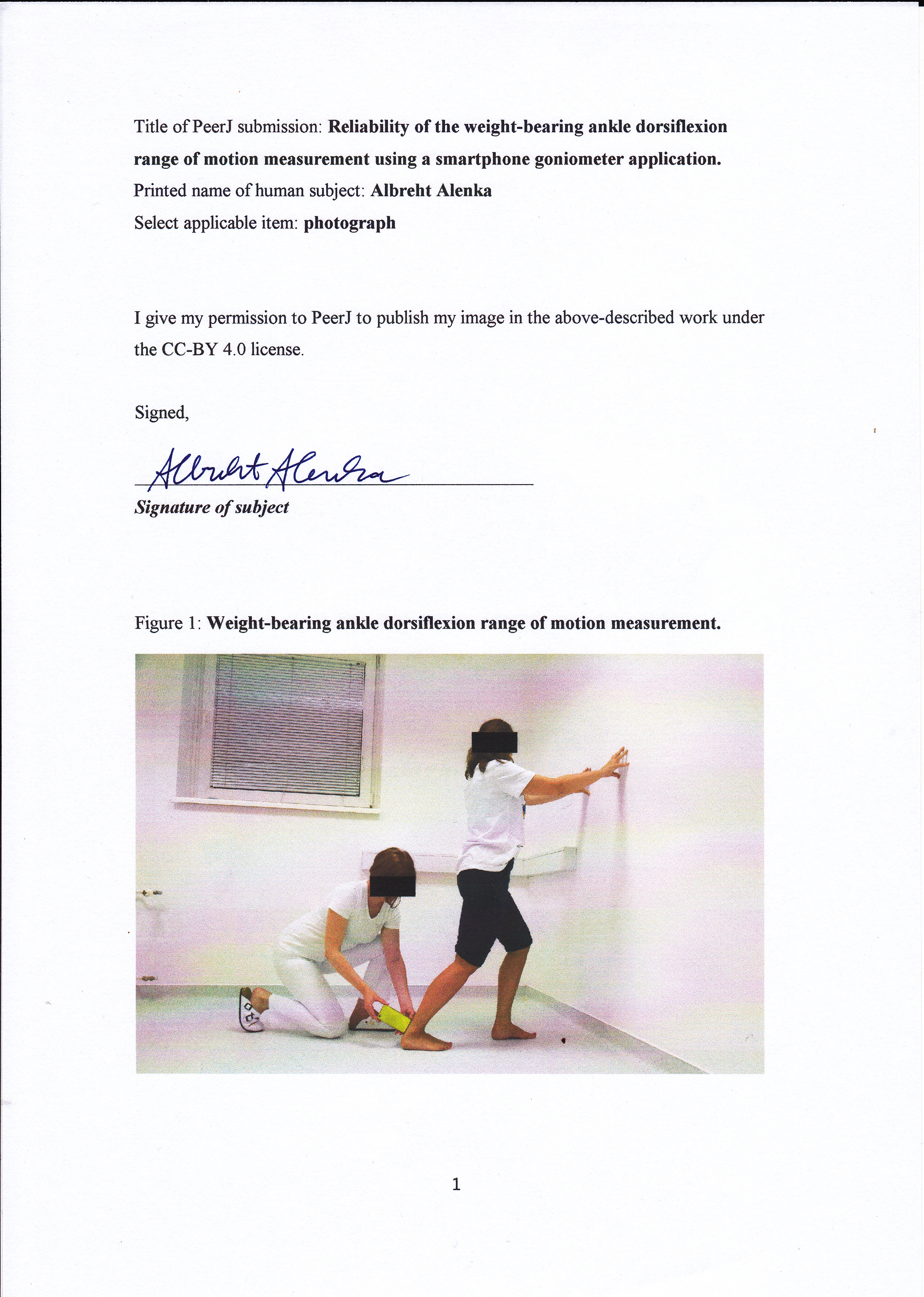

Supplement: Supplemental Information 2 [file peerj-09-11977-s002.png]
